# Impact of Antioxidant and Anti-Inflammatory Functions of HDL in Diseases—2nd Edition

**DOI:** 10.3390/antiox14030358

**Published:** 2025-03-18

**Authors:** Carlo Cervellati, Judit Marsillach

**Affiliations:** 1 Department of Morphology, Surgery and Experimental Medicine, University of Ferrara, 44121 Ferrara, Italy; 2 Department of Environmental & Occupational Health Sciences, University of Washington, Seattle, WA 98105, USA; jmarsi@uw.edu

In recent decades, significant advancements in lipidology have profoundly reshaped our understanding of the biological roles of lipids and lipoproteins, particularly high-density lipoproteins (HDLs). The traditional view of HDLs as merely transporters of cholesterol from peripheral tissues, including macrophages involved in the atherosclerotic process, to the liver (known as Reverse Cholesterol Transport, RCT) has evolved into a more comprehensive understanding that captures the multifaceted and diverse nature of these lipoproteins [1,2,3]. HDLs are now recognized as a complex and heterogeneous family of particles that vary in size, density, and function [4]. This diversity is substantial, with most particle subspecies having pronounced athero-protective functions, while others may exert pro-atherogenic activity [3]. This paradoxical, harmful action of HDLs is primarily due to the increased susceptibility of the lipoprotein to oxidative modification, which, similar to LDLs, causes significant structural and functional changes [2].

Converging reports indicate that smaller HDL particles possess the highest protective activity. In line with this, the prospective study by Stadler et al. [5], conducted on patients with chronic kidney disease, found that the levels of cholesterol Apolipoprotein A1 (ApoA1) and ApoA2 were independently associated with an increased risk of all-cause mortality.

The fact that ApoA1 may be used as prognostic (as well as diagnostic) biomarkers for multiple disease states other than cardiovascular diseases (CVDs) has been widely documented [6]. This is mainly due to the ability of this main protein constituent of HDL to coordinate and modulate a wide range of lipoprotein properties, including promoting cholesterol efflux from macrophages, providing antioxidant and anti-inflammatory actions, detoxifying xenobiotics, and preserving endothelial integrity and function, among others [7,8]. The pivotal role of ApoA1 is confirmed in Cho’s study. The authors first demonstrated that a mixture of lipid-free ApoA1 and CIGB-258, a peptide with proven anti-inflammatory and antioxidant activity, synergistically improves the protective functions of human HDLs [8]. They then found that in zebrafish treated with the highly toxic carboxymethyllysine, this combination has wound-healing functions and increased survival rates by lowering reactive species formation and down-modulating pro-inflammatory cytokines [8].

As stated above, ApoA1 is fundamental for the functionality of HDL. However, this apolipoprotein does not act alone but through mutual interaction with other so-called accessory proteins, such as Paraoxonase 1 and 3 (PON-1 and -3), Glutathione peroxidase 1, Lipoprotein-lipase A2, etc. [2,9]. In particular, PON1 has been widely suggested to be the main partner of ApoA1 in driving the antioxidant activity of HDL [10]. This feature accounts for the substantial body of evidence linking PON-1 to the onset and progression of diseases where oxidative stress plays a significant pathogenic role, including cardiovascular diseases (CVDs) and metabolic disorders [11,12,13]. This aligns with the review by Denimal [14], which meticulously describes the mechanisms by which impaired antioxidant and anti-inflammatory activity of HDL may contribute to Type II Diabetes (T2D) and its related complications, while the link with Type I Diabetes (T1D) is not yet fully established. The review also highlights the role of PON1 in counteracting LDL oxidation in diabetes, a key event that predisposes these patients to a high risk of cardiovascular diseases (CVDs). Additionally, it discusses the potential of PON1 as a pharmacological target in T2M [14].

Obesity is a well-known risk factor for Type II Diabetes (T2D) [15]. Consistently, Castane and colleagues demonstrated that morbidly obese individuals have lower enzymatic activities of PON1 compared to non-obese individuals [16]. In line with previous reports [17], PON-arylesterase and PON-paraoxonase activities were inversely related to anthropometric parameters of central and overall obesity. Additionally, PON1 concentration was found to inversely correlate with the degree of hepatic steatosis [16].

PON activities can be measured in biological fluids using simple and cost-effective spectrophotometric assays [18]. This has prompted numerous biomedical researchers worldwide to conduct population studies investigating the potential of PON1 as a diagnostic and prognostic biomarker. Significant contributions in this area were provided by two studies published in this Special Issue.

In the first work, Stankovic et al. found that blood levels of PON1, along with other protein and lipid components of HDL, may be used in pregnant women to predict the development of typical cardiometabolic complications, such as gestational diabetes mellitus and hypertensive disorders of pregnancy [19]. PON1, ApoM, ApoA1, and Serum Amyloid A (accessory proteins that impair HDL functionality) were found to be altered to varying degrees in the first, second, or third trimesters in pregnant women with complications compared to those without. The most striking and applicable finding was the independent association of Sphingosine-1-phosphate with cardiometabolic pregnancy complications in the first trimester of pregnancy [19]. This suggests that Sphingosine-1-phosphate may be an early predictive biomarker for these clinical complications.

In the second study on the potential of PON-1 as a disease biomarker, Trentini et al. evaluated the arylesterase activity levels and the concentration of another HDL-associated protein from the same family in 99 Alzheimer’s disease (AD) patients, 100 patients with mild cognitive impairment (MCI), and 79 cognitively normal controls [20]. Consistent with previous findings, they observed a significant decrease in arylesterase activity in both the MCI and AD patients compared to the controls, while the PON3 levels remained unchanged. Interestingly, PON3 showed a strong association with the pro-oxidant myeloperoxidase, suggesting a preferential physical association of PON3 with dysfunctional HDL.

The studies reviewed so far clearly indicate that proteins like PON1, which contribute to the antioxidant and anti-inflammatory properties of HDL, have great potential as pharmacological targets in various diseases. Unfortunately, no effective pharmacological or natural approaches in this field have been officially approved and commercialized yet. The study by Cho et al. provided compelling pre-clinical evidence that could help address this issue [21]. Specifically, it assessed the effect of ozonated sunflower oil (OSO) on various metabolic parameters, including PON1 activity, in rats. The most striking findings of this investigation were that OSO supplementation stabilized apoA-I/HDL and increased HDL-(PON)-1 activity. Moreover, a microinjection of plasma obtained from rats treated with OSO rescued zebrafish embryos and adults from carboxymethyllysine (CML)-induced toxicity and prevented CML-induced hepatic damage, fatty liver changes, oxidative stress, and systemic inflammation in zebrafish [21].
